# Paediatricians’ Views on Pain in Children with Profound Intellectual and Multiple Disabilities

**DOI:** 10.3390/brainsci11030408

**Published:** 2021-03-23

**Authors:** Lucie Petigas, Christopher J. Newman

**Affiliations:** 1 Faculty of Biology and Medicine, University of Lausanne, 1011 Lausanne, Switzerland; Lucie.Petigas@unil.ch; 2 Paediatric Neurology and Neurorehabilitation Unit, Lausanne University Hospital and University of Lausanne, 1011 Lausanne, Switzerland

**Keywords:** child, disability, pain, paediatrician, profound intellectual and multiple disabilities, survey

## Abstract

Pain is a frequent issue in children with profound intellectual and multiple disabilities (PIMD). Its identification and treatment can prove highly challenging for primary care physicians, mainly because of the children’s limited communication abilities. We used an online survey to explore paediatricians’ views regarding the experience and management of pain in children with PIMD and invited 480 professionals working in the canton of Vaud, Switzerland, to take part. We received 121 responses (participation rate 25.5%). A large majority of respondents provided care to children with PIMD. All paediatricians considered that these children feel pain at least as much as typically developing children. However, paediatricians had mixed views on their tolerance to pain. More than 90% held the view that their pain is under-assessed and undertreated. The principal barriers they reported to appropriate management were communication limitations with the child, difficulties in pain assessment, lack of knowledge about children with disabilities and lack of experience. Paediatricians have complex opinions regarding how children with PIMD experience pain and how to manage this problem. Professional education and training on the specificities of children with PIMD, including how to address their pain, seem necessary to foster paediatricians’ ability and confidence in approaching this complex issue.

## 1. Introduction

The prevalence of children with profound intellectual and multiple disabilities (PIMD) is estimated to be between 0.4 and 1.3‰ [1,2]. PIMD is defined by the association of (1) a severe to profound intellectual disability with (2) a significant motor impairment leading to (3) complex health care needs and high dependency in daily activities [2]. Furthermore, individuals with PIMD have extremely delayed intellectual and social functioning, with little or no apparent understanding of verbal language and little or no symbolic interaction with objects. Therefore, standard pain self-assessment is usually compromised, and the challenge of communicating with these children can lead to major difficulties in pain evaluation by their healthcare providers, contributing to inefficient pain management [3,4].

The International Association for the Study of Pain recently revised its definition of pain as “an unpleasant sensory and emotional experience associated with, or resembling that associated with, actual or potential tissue damage” [5]. This definition accounts for the subjective nature of pain and does not equate pain with self-reporting or the ability to self-report. Therefore, it also applies to children with neurological disabilities who are unable to express their pain in words. The definition acknowledges that the pain experience integrates both sensory and emotional components, with the brain supporting a complex interaction between nociception, the feeling of unpleasantness and emotional states such as distress, anxiety or fear [6]. Pain can impact all areas of life, including sleep, mood and interactions with others, but also physical and cognitive abilities [7]. This is even more important in children with PIMD, for whom pain can significantly worsen the major functional limitations associated with their neurological condition. Considering that children with PIMD are at a higher risk of experiencing pain due to their medical complexity [8], efficient pain identification and management are essential in this population. Common sources of nociceptive pain in PIMD are skeletal (hip subluxation, scoliosis, osteopenia and pathological fractures), related to neuromotor disorders (spasticity, dystonia, dyskinesia and immobility), digestive (gastroesophageal reflux, constipation and dysmotility) and iatrogenic (investigations, surgery and physiotherapy) [9,10]. Problems with mobility and posture may lead to pressure sores or distortion of internal organs. For certain children with PIMD, even habitual daily care activities, such as dressing, may be associated with significant pain [8]. In addition, central nervous system processes contribute to amplify their pain experience, notably central sensitization, neuropathic pain and autonomic dysfunction [10]. In one cohort study, half of the children with severe cognitive impairment had pain at least weekly, with an average duration of nine hours per week [11]. Half of those in another registry-based study of children with severe cerebral palsy regularly experienced pain, primarily musculoskeletal pain [12]. Even young children have significant pain. In a cross-sectional study of children with cerebral palsy aged 5 to 10 years, pain was present in 52% of the children at all severity levels, restricting their daily lives, especially sleep, schoolwork and being with friends [13].

Long-held beliefs among parents and physicians of children with PIMD suggest that individuals with intellectual disabilities have an impaired perception of pain. Several studies have refuted this suggestion [14]. However, these beliefs seem to persist in clinical practice and can be part of why children with PIMD may be at a higher risk of poor pain management [3]. Valkenburg et al. [15] surveyed Dutch anaesthesiologists, specifically focusing on their management of perioperative pain and their knowledge of pain assessments for non-communicating children, and explored their beliefs about these children’s subjective experience of pain. A majority thought that disabled children were not more sensitive to pain and did not require more analgesia than typically developing children. Very few were aware of pain scales specifically developed for children with limited communication abilities.

There are few studies in children with PIMD regarding pain assessment (mostly regarding clinical assessment tools) and pain management, and these have rarely explored the opinions and attitudes of healthcare professionals [4,15,16]. None have specifically included primary care physicians who are at the forefront of pain identification and management within the healthcare system. Consequently, our study aimed to (1) explore paediatricians’ views regarding the experience and management of pain in children with PIMD and (2) investigate whether paediatricians’ demographic and professional characteristics were associated with their views of pain in children with PIMD.

## 2. Materials and Methods

### 2.1. Design

We conducted a cross-sectional descriptive study using an online survey.

### 2.2. Population

We invited all professionally active physicians working in general paediatrics and paediatric subspecialties in the canton of Vaud, Switzerland (population 800,162 inhabitants), to take part. Undergraduate medical students were excluded. We invited a total of 480 professionals, 177 paediatricians working in community practice and 303 paediatricians working in hospitals. Nineteen professionals worked in both settings.

### 2.3. Survey Development and Content

We used Valkenburg et al.’s questionnaire to develop our survey [15]. We completed the questionnaire with questions extracted from the thematic analysis of the transcribed outputs of five semi-structured interviews with key stakeholders. These stakeholders included a physiotherapist, a nurse working in a special needs school, a nurse who specialized in palliative care, a hospital paediatrician and a representative of a parents’ association. These interviews focused, from a multi-professional perspective, on the challenges and difficulties in pain care for children with PIMD and on the resources at the disposal of each interviewee. The five concurring themes that emerged from the analysis were (1) the experience of pain in children with PIMD, (2) assessing their pain, (3) managing their pain, (4) barriers to providing them with appropriate medical care and (5) barriers to treating their pain with medication.

The survey explored (1) eight demographic and professional characteristics of the participants (gender, age, place of employment, country of origin, country of study, the field of current paediatric activity, years of experience in paediatrics, years of experience in current activity), (2) professional exposure to children with PIMD, (3) physicians’ opinions on the pain experience of children with PIMD and (4) physicians’ pain assessment and management of children with PIMD. Each item was formulated as a statement, with agreement versus disagreement toward the statement graded on a four- to five-point Likert scale (with an additional neutral response for knowledge-based questions).

Before starting data collection, the questionnaire was submitted to two hospital-based paediatricians working in the field of paediatric disability, who independently validated its content.

### 2.4. Data Collection

The survey was anonymous, in French and made available online via Google forms. Paediatricians working in community practices were contacted through the mailing list of their professional association (Groupement des Pédiatres Vaudois) and paediatricians working in hospitals through the heads of paediatric departments of the University Hospital (CHUV, Lausanne) and five regional hospitals (Aigle, Vevey, Morges, Nyon, Yverdon).

Participants received an email invitation to take part in the survey. Response to the survey implied consent. Data collection began in May 2019. We sent two reminders with a delay of three weeks in between, and the survey was closed one month after the last reply.

Because the study included healthcare professionals and was strictly anonymous, formal agreement by the regional ethics commission was waived, according to the Swiss Federal Act on Research Involving Human Beings, art. 2.2 al. c [17].

### 2.5. Data Analysis

Data were analysed descriptively by counts and proportions. For data analysis, given our sample size, responses were grouped into two to three categories (e.g., disagree, agree and an additional neutral response for the knowledge-based questions). For demographic and professional characteristics, age, experience and time in current activity were categorised in 5- to 10-year ranges. Countries of origin and study were classified between Switzerland, Europe or other. Current activity was classified between general paediatricians, neuropaediatricians (including neurologists, neurorehabilitation specialists and developmental paediatricians) and other paediatric specialists (for a complete overview of specialities, see question 5 in Supplementary Materials Table S1). Based on the notion of consensus in Delphi processes [18], we then classified the responses as consensual, when ≥80% of participants provided the same response, and non-consensual, when <80% of participants provided the same response. For non-consensual responses, we performed association analyses between demographic and professional characteristics and the responses of the participants using cross-tabulation and chi-square tests. After applying the Bonferroni correction to decrease the risk of false positive significance and fortuitous findings due to multiple comparisons, *p* ≤ 0.0063 was considered significant (*p*-value of 0.05 corrected by the eight demographic and professional characteristics). We used SPSS Statistics v.25 (IBM, Armonk, NY, USA) to perform the analyses.

## 3. Results

### 3.1. Survey Population

The participation rate was 25.5% (121 responses out of 480 invites). There were no missing data in the responses. There was no significant difference in response rates (χ^2^(1480) = 1.01, *p* = 0.31) between paediatricians working in hospitals (26.7%) and those working in the community (22.6%). The demographic and professional characteristics of the respondents are reported in Table 1. A large majority of the respondents (96.8%) reported taking care of children with PIMD in their practice, more than half regularly, that is, at least once per month (51.2%).

### 3.2. Survey Responses

#### 3.2.1. The Experience of Pain in Children with PIMD

**Figure 1:**
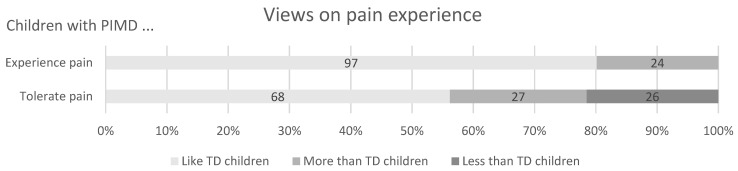
Paediatricians’ (*n* = 121) views on the pain experience of children with profound intellectual and multiple disabilities (PIMD) compared with typically developing (TD) children.

A majority of paediatricians considered that children with PIMD experienced pain in the same way as typically developing children and 20% considered that the experience of pain was higher for children with PIMD. Tolerance to pain was judged as higher in children with PIMD by a quarter of respondents and lower by a fifth (Figure 1).

If most participants (94.2%) responded that there was no correlation between the severity of disability and sensitivity to pain, most (90.1%) agreed that the more severe the disability, the higher the difficulty in distinguishing pain from other negative emotions. A majority of paediatricians (78.5%) agreed that each pain experience included an emotional response in children with PIMD, and a slim majority (52%) responded that the emotional response to pain of children with PIMD differed from that of typically developing children.

#### 3.2.2. Pain Assessment

A large majority of respondents (95%) agreed that the pain of children with PIMD was under-evaluated. Around half (52.1%) answered that the use of pain scales was more important than with typically developing children, and around the other half (46.3%) answered that the use of pain scales was as important as with typically developing children.

Around half of the participants (49.6%) were familiar with the scales validated for the assessment of pain in children with PIMD. The most known scales were the Face, Legs, Activity, Cry, Consolability (FLACC) scale [19] (44.6%) and the Douleur Enfant San Salvadour scale [20] (36.4%). These were also the most used (respectively, 34.7% and 24.8%). More than half (51.2%) of the paediatricians did not use pain scales for these children.

#### 3.2.3. Pain Management

The vast majority of respondents reported that pain was undertreated in children with PIMD, and a minority reported that it was overtreated. More than half of the respondents considered pain to be more difficult to relieve in children with PIMD.

Regarding analgesics, two-thirds responded that children with PIMD needed them more frequently, and opinions were mixed about their effectiveness compared to typically developing children.

**Figure 2:**
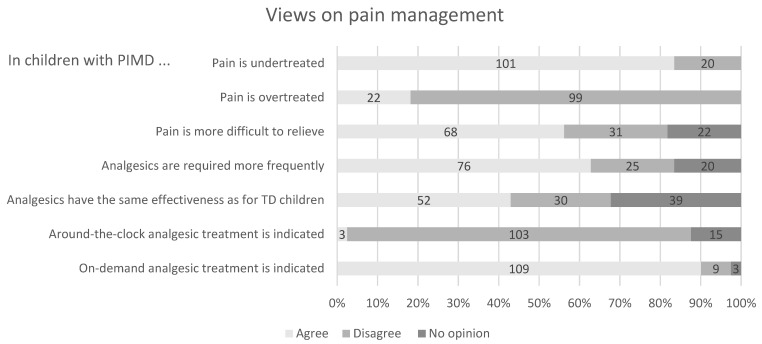
Paediatricians’ (*n* = 121) views on pain management of children with PIMD.

Most of the participants disagreed with prescribing a round-the-clock analgesic treatment, but a large majority responded that an on-demand treatment should be prescribed for suspected pain without the need for additional medical guidance (Figure 2).

#### 3.2.4. Barriers to the Appropriate Medical Care of Children with PIMD

Paediatricians reported that the main barriers to appropriate clinical management of children with PIMD were limited communication with the child (84.2%), difficulties in pain assessment (82.6%), lack of personal experience (71.9%) and lack of knowledge about disabled children (71.9%). Less than half of the participants (47.1%) reported a lack of knowledge about pain as a barrier to the adequate care of children with PIMD.

#### 3.2.5. Barriers to Treating Pain with Medication in Children with PIMD

Two-thirds of respondents (68.5%) considered the possibility of masking the source of pain with analgesics to be a potential barrier to treating pain with medication. Conversely, a minority of respondents considered the risk of addiction to analgesics (17.4%), the fear of adverse effects of opioids (28.1%), parental pressure (27.3%) and the fact that treatment could hinder the investigation of potential sources of pain (33.1%) as significant barriers to analgesic treatment. Respondents had mixed views about the fear of respiratory depression with opioids, with less than half (46.2%) considering this as a barrier to treatment.

#### 3.2.6. Association Analyses of Non-Consensual Responses

Non-consensual views were present within all five themes of the survey: for experience of pain, 3/6 of the questions had non-consensual responses; for assessment of pain, 2/3; for management of pain, 3/7; for barriers to providing appropriate medical care, 3/5; and for barriers to treatment with analgesics, 6/7. Only certain professional characteristics demonstrated significant associations with these non-consensual responses.

Neuropaediatricians were significantly more likely to agree that pain was over-treated in children with PIMD (9/22 of neuropaediatricians agreed compared with 12/99 of other paediatricians, χ^2^(1121) = 10.4, *p* = 0.001).

General paediatricians were more likely to agree that pain might be masked by analgesic treatment than neuropaediatricians and other paediatric specialists (34/87 of general paediatricians agreed compared with 3/31 of paediatric specialists, χ^2^(1121) = 10.5, *p* = 0.001).

Paediatricians working outside hospitals were more likely to agree that the fear of the adverse effects of opioids could be a barrier to adequate pain treatment in children (18/40 of community paediatricians agreed compared with 17/81 of hospital paediatricians, χ^2^(1121) = 7.5, *p* = 0.006).

## 4. Discussion

Our study suggests that paediatricians, a large majority of who provide care to children with PIMD, have complex opinions regarding how these children experience pain and how to manage this issue. These views were largely unrelated to the physicians’ demographic and professional characteristics, with a few exceptions.

Despite longstanding beliefs about pain insensitivity or indifference in persons with intellectual disabilities [21,22], none of the respondents believed that children with PIMD experienced pain less than typically developing children. Our findings are in line with those of Valkenburg et al. [15], who found that around 60% of anaesthetists considered children with intellectual disabilities equally sensitive and 30% considered them more sensitive to pain than typically developing children. Limited research in children with neurodevelopmental disabilities has demonstrated that their pain thresholds are similar to [23], if not lower than [24], those of children with typical development. This is possibly related to alterations in somatosensory processing associated with cerebral impairment [25]. However, paediatricians had more mixed views on the way children with PIMD modulate their experience and expression of pain, with a lack of consensus on how they tolerate pain. Whether children with severe cognitive limitations can present adaptive coping to pain is largely undetermined, with a lack of knowledge on the resilience factors, both personal and environmental, that could influence the lived experience of pain in this population [26]. Children with intellectual disabilities experience more pain and anxiety during needle-related procedures than typically developing children [27]. Additionally, recent research by near-infrared spectroscopy has shown that children with severe to profound intellectual disabilities demonstrate cortical activation during venepuncture that typically developing children do not [28]. This suggests that children with PIMD go through a different subjective experience of pain compared with their cognitively unimpaired peers and that this different experience may be related to distress in children who are unable to rationalise unfolding events related to painful procedures. Beyond the biological and psychological determinants of pain, it is likely that social determinants, such as the attitudes of parents or other caregivers, also modulate the experience and expression of pain, as conceptualized in Craig’s social communication model of pain [29].

Paediatricians mostly acknowledged the emotional component of pain in children with PIMD beyond pure nociception; however, half thought that it differed from the emotional experience of children with typical development. Distinct neural systems support the pain experience. The lateral pain system supports the sensory-discriminative dimension of pain by identifying the characteristics of painful stimuli. The medial pain system underpins the affective-motivational dimension of pain, which is most closely associated with emotion and is the core of the unpleasantness generated by nociception. A third aspect, strongly intertwined with both previous dimensions, is the cognitive dimension under higher cortical control, which influences the appreciation and understanding of pain [6]. One can reasonably hypothesise that abnormal cerebral function and severe cognitive impairment in children with PIMD can alter the experience of pain, especially in its higher processing. However, their communication difficulties limit our ability to verify this hypothesis.

Paediatricians largely held the view that pain was under-assessed and undertreated in children with PIMD. This concurs with a previous study exploring the attitudes of clinicians regarding pain and cognitive impairment in a large children’s hospital, where 99% of physicians encountered difficulties in pain assessment and 71% of all clinicians reported that analgesics were under-prescribed for these children [4]. Although a large majority of our sample estimated that the use of validated pain assessments was at least as important for children with typical development, only half of the respondents knew and used these pain scales in their clinical practice. Lack of familiarity and awareness of available tools, organizational and time constraints and the absence of a clear consensus as to which scales should be used in routine practice are all factors that can act as barriers to the adequate implementation of pain scales in daily practice [14]. Moreover, there is evidence that health professionals experience a lack of confidence in this type of care despite the existence of valid tools [30]. Our proportion of users is a major improvement on previous figures, with only 1 in 55 paediatric wards in North-Eastern Italian hospitals using pain scales adapted to children with cognitive impairments in 2011 [31]. Similarly, only 4% of child anaesthetists reported the use of a pain assessment tool validated for children with severe neurological impairments in 2019 [15]. A few behavioural pain assessment tools have been validated in this population. Most of them rely on documentation of baseline childhood behaviour with regular caregivers to detect further changes of behaviour that could prove indicative of pain and allow the inclusion of less typical pain behaviours, such as increased muscle tone, self-injury or laughing [32].

Despite reporting a tendency to undertreat children with PIMD, a majority of respondents believed they needed analgesics more frequently than typically developing children do, and a large majority warranted on-demand analgesic treatment for this population. However, very few advocated for round-the-clock treatment, and less than half of the respondents believed that analgesics had the same effectiveness in children with PIMD as in typically developing children. This contrasts with the opinions of anaesthetists, among whom only a small minority believed that children with intellectual disabilities required more analgesia [15]. However, these opinions specifically addressed peri-procedural pain. Paediatricians considered analgesics to be less effective in children with PIMD, but favouring on-demand administration may have contributed to this perception. The contrasting, if not paradoxical, views of paediatricians, who generally considered children with PIMD as undertreated but refrained from prescribing round-the-clock medication, reflects the perceived complexity of providing analgesia to these children. Not only is this related to the difficulty in identifying the presence, source and extent of pain but also because several pain behaviours in children with PIMD are compounded by an imbalance between inhibitory and excitatory signals treated by the central nervous system within the context of their impaired cerebral function. Certain children ultimately present opioid-resistant pain behaviours, especially in the case of recurrent or chronic pain, and adapted pain ladders (e.g., the “neuro-pain” ladder), including the early use of gabapentinoids, have been suggested for this population [33]. Lack of familiarity with such guidelines, added to the complexity of these children’s conditions, may further contribute to paediatricians’ difficulties in providing effective analgesia. Indeed, features such as muscle weakness, co-morbidities, including malnutrition or respiratory insufficiency, and concomitant medication or even polypharmacy must all be considered when prescribing analgesic medication for children with PIMD because these can significantly interfere with dosages and duration of treatment and contribute to side effects [34].

More than 40% of neuropaediatricians, significantly more than the 12% of other paediatricians, reported a tendency to overtreat pain in children with PIMD. This could reflect the view among certain neurological specialists that a proportion of behaviours attributed to pain that children with PIMD exhibit are inappropriately treated by opioid prescription. First, they may consider that opioids are not the medication of choice as an early line of treatment, especially for chronic pain in children with PIMD. Second, these specialists have more experience with children with neurological disabilities and have a stronger knowledge of the variety and complexity of behavioural issues and expressions that accompany severe to profound intellectual disability. They may therefore be more attuned to distinguishing expressions of pain from similar behaviours caused by other stressors or by their cerebral impairment.

The major issues reported by paediatricians in providing appropriate medical care to children with PIMD were their lack of knowledge about disability, their limited experience with disabled children, and difficulties in communicating with non-verbal children and in evaluating their pain. Weaknesses in the training of physicians about cognitive and physical disabilities in children have been reported previously [35,36,37]. Up to three-quarters estimated that they were not sufficiently trained to confidently care for these children, most of their knowledge being acquired informally through on-the-job training and through mentorship. If children with PIMD have strongly limited communication capabilities, with a restricted, if not absent, ability to self-report pain, physicians should always explore limited self-reporting as a possible means of collecting pain information [21]. Pain may also prove difficult to distinguish from other conditions, such as distress, anger, sadness or anxiety, even for familiar caregivers, with an overlap in expressed behaviours [38]. Less than half of paediatricians reported a lack of knowledge about pain as a barrier to the management of children with PIMD. Around 80% of our sample received their medical education in Switzerland, where pain is one of the generic situations that all physicians are expected to be able to manage on day one of their residency after graduating from medical school [39].

The principal barriers to prescribing pain medication in children with PIMD reported by paediatricians were the fear of masking the source of pain, followed by the risk of respiratory depression linked to opioids and the concern that symptomatic medication could slow or hinder the correct investigation of pain. The fear that effective pain treatment will mask pain in children with limited communication abilities, especially from a new source of pain, is common among prescribers [32]. However, a case series of children with severe neurological impairments showed that while they were being treated efficiently for recurrent pain behaviours, the onset of new pain behaviours allowed the diagnosis of new sources of pain, such as urinary tract infections [40]. Likewise, there is no evidence of a decrease in the etiological investigations of pain in children with disabilities who receive appropriate pain medication. The concern of masking pain was significantly more prevalent among general paediatricians, possibly because they intervene earlier and have a more global view of the clinical workup of pain behaviours than speciality paediatricians do. The fear of missing a diagnosis that could be detected if pain were not masked by analgesics may be higher for first-line professionals who are in charge of the global healthcare of children with PIMD, especially since the expression of pain is a problem for these children. This may contribute to the conservative attitude of paediatricians who, as discussed above, seem to refrain from prescribing round-the-clock analgesia, favouring on-demand treatment. Fear of respiratory depression linked to opioids is a common concern among health professionals who care for children. Multiple studies have shown that opioids are effective and safe in children when they are appropriately introduced and titrated appropriately [41]. However, children with PIMD may prove more challenging due to associated risk factors, such as poor airway control or hypoventilation [21]. This concern was significantly higher among community-based paediatricians. They are far less likely to prescribe opioids in their practice than hospital-based professionals, do not have direct access to the multidisciplinary resources of a hospital, such as pain teams, and are less familiar with the processes that allow for the secure prescription and follow-up of opioid treatments.

Our study’s main limitation is that we cannot exclude a degree of responder bias with a 25% participation rate. However, the age and gender structure of our sample was representative of paediatricians, both within our recruitment area and at the national level [42]. We were not able to control further for non-responders for whom we had no personal or professional data. Our sample size and the size of certain subgroups of participants may have limited our ability to detect certain associations between personal characteristics and non-consensual responses. This study was based on self-reported practice, and it is impossible to know how well our results reflect actual practice. Further observational studies on clinical assessment and management practices in this population are needed. Finally, we performed the study in a single region, and our results may not reflect wider variations in paediatricians’ views on this subject at the international level.

## 5. Conclusions

Paediatricians’ views and beliefs about the pain of children with PIMD are equivocal and complex. Caring for children with PIMD entails navigating uncertainty, and previous qualitative research has shown that this erodes professional confidence due to perceived deficits in both skill and knowledge sets [43]. A large part of our respondents acknowledged their difficulties and limits in assessing and managing pain in this group of children but had a genuine willingness to do well. Half of our sample showed some familiarity with dedicated pain scales. Professional education and training on the specificities of children with PIMD, including how to address their pain, seem necessary to foster their ability and confidence in approaching this complex issue. Ideally, this training should be grounded in evidence. This still demands strengthening given the relatively scarce research into pain in this population, which is often excluded from pain studies because of functional limitations, methodological issues surrounding pain ascertainment and measurement and the difficulty in achieving appropriate sample sizes for exploratory and interventional studies. We believe that by furthering both professional education and research into pain in children with PIMD, the quality of their care and, ultimately, their quality of life will improve.

## Figures and Tables

**Table 1:** Personal characteristics of survey respondents.

Demographic and Professional Characteristics *n* (%)						
Gender	Male 39 (32.2%)			Female 82 (67.8%)		
Age range (years)	25–34	35–44		45–54		>55
	39 (32.2%)	36 (29.8%)		31 (25.9%)		15 (12.4%)
Country of origin	Switzerland		Europe		Other	
	89 (73.5%)		29 (23.9%)		5 (4.1%)	
Country of study	Switzerland		Europe		Other	
	96 (79.3%)		22 (18.2%)		4 (3.3%)	
Current activity	General paediatricians		Neuropaediatricians		Other paediatric specialists	
	87 (71.9%)		12 (9.9%)		22 (18.2%)	
Experience in paediatrics (years)	0–5	6–10		11–20		>20
	33 (27.3%)	17 (14%)		44 (36.4%)		27 (22.3%)
Current activity (years)	0–5	6–10		11–20		>20
	52 (43%)	30 (24.8%)		28 (23.1%)		11 (9.1%)
Place of work	Hospital			Community		
	81 (66.9%)			40 (33.1%)		

## Data Availability

The data presented in this study are available on request from the corresponding author.

## Supplementary Materials

The following are available online at https://www.mdpi.com/2076-3425/11/3/408/s1, Table S1: Survey script.
