# Supplementary material for: Paediatricians’ Views on Pain in Children with Profound Intellectual and Multiple Disabilities
Source: Brain Sci. 2021 Mar 23;11(3):408. doi: 10.3390/brainsci11030408 (PMC8004709; doi:10.3390/brainsci11030408)
Supplement: Supplementary file 1 [file brainsci-11-00408-s001.pdf]

Table S1. Survey script

Paediatricians' attitudes towards pain in non-communicating children with profound intellectual and multiple disabilities

1. What is your gender?
   a. Female
   b. Male

2. How old are you ? n years

3. What is your home country ? dropdown list

4. What was your country of medical studies ? dropdown list

5. In which field of paediatrics are you currently working ? (more than one answer possible)
   a. General paediatrics
   b. Paediatric emergency medicine
   c. Intensive care
   d. Neonatology
   e. Endocrinology and diabetology
   f. Gastroenterology
   g. Cardiology
   h. Pneumology
   i. Nephrology
   j. Neurology
   k. Neurorehabilitation
   l. Developmental paediatrics
   m. Immunology, allergology & rheumatology
   n. Onco-hematology
   o. Infectiology
   p. School medicine

6. How many years of experience do you have in paediatrics? n years

7. How many years of experience do you have in your current activity? n years

8. Where do you work? (more than one answer possible)
   a. University Hospital
   b. Regional Hospital
   c. Community practice
   d. School
   e. Research

Children with profound intellectual and multiple disabilities in my practice

This study focuses on children with profound intellectual and multiple disabilities (PIMD). PIMD is a term that refers to an heterogenous population of children with a motor disability combined with a severe or profound intellectural disability, resulting in an extreme restriction of autonomy and opportunities for perception, expression and relationship*. The disability's origin is variable, covering multiple etiologies (severe cerebral palsy, dysmorphogenetic syndromes, neurometabolic disorders, etc.). In this study we are specifically interested in the severe communication limitations of these children, described as non-communication or pauci-communication due to absence of verbal expression associated with a significant delay in understanding language. The objective is to explore to which difficulties in identifying and managing their pain this could lead for the physicians taking care of this particular children.

** According to the annex XXIV ter of the National Technical Center of Studies and Research about Handicap and Disabilities. (CTNERH, Centre Technique National d'Etudes et de Recherche sur les Handicaps et les Inadaptations), 29.10.1989.*

For more informations about PIMD and if needed to better recognize these children in your practice : https://www.perce-neige.org/actus/comprendre-le-handicap/le-polyhandicap/

9. Do you see children with PIMD in your practice?
    a. Yes
    b. No

10. I see children with PIMD in my practice …
    a. Once to a few times per week
    b. Once to a few times per month
    c. A few times per year
    d. Once or twice per year
    e. Less than once per year
    f. Never

The experience of pain in children with PIMD

This section of the survey explores your perceptions and opinions about the pain experience of children with PIMD. We are aware that each child experiences pain differently and that it is complex to generalize. However, our goal is to explore your opinion and not your theoretical knowledge. Please answer in the way that best corresponds to your opinion.

11. Children with PIMD experience pain…
    a. More than ordinary children.
    b. As ordinary children.
    c. Less than ordinary children.

12. Children with PIMD tolerate pain…
    a. More than ordinary children.
    b. As ordinary children.
    c. Less than ordinary children.

13. The more the child's disability is important the less he is sensitive to pain.
    a. Totally agree
    b. Agree
    c. Disagree
    d. Totally disagree
    e. Don't know

14. The more the child's disability is important, the more difficult pain is to differentiate from other emotions.
    a. Totally agree
    b. Agree
    c. Disagree
    d. Totally disagree
    e. Don't know

15. Every pain experience leads to an emotional reaction in disabled children.
    a. Totally agree
    b. Agree
    c. Disagree
    d. Totally disagree
    e. Don't know

16. The emotional component of pain…
    a. Is the same for children with PIMD as for typically developing children
    b. Differs between children with PIMD and typically developing children
    c. Is absent in children with PIMD

Pain assessment in PIMD

17. Pain is under evaluated in children with PIMD.
    a. Totally agree
    b. Agree
    c. Disagree
    d. Totally disagree

18. Do you know any pain scales adapted to children with PIMD?
    a. Yes
    b. No

19. Which of these scales do you know about?
    a. San Salvadour Pain Scale
    b. Non-Communicating Children's Pain Checklist (NCCPC)
    c. Face Legs Activity Cry Consolability scale (FLACC)
    d. None

20. Which of these scales do you use?
    a. San Salvadour Pain Scale
    b. Non-Communicating Children's Pain Checklist (NCCPC)
    c. Face Legs Activity Cry Consolability scale (FLACC)
    d. None

21. In the care of disabled children, the use of a pain scale is…
    a. More important than with ordinary children.
    b. As important as with ordinary children.
    c. Less important than with ordinary children.
    d. Not important.

Pain management in PIMD

22. Children with PIMD need analgesic more frequently than typically developing children.
    a. Totally agree
    b. Agree
    c. Disagree
    d. Totally disagree
    e. Don't know

23. Analgesics' efficacy is the same in children with PIMD in typically developing children.
    a. Totally agree
    b. Agree
    c. Disagree
    d. Totally disagree
    e. Don't know

24. Children with PIMD should have around-the-clock analgesics in their daily treatments.
    a. Totally agree
    b. Agree
    c. Disagree
    d. Totally disagree
    e. Don't know

25. Disabled children should have on-demand analgesics that their usual care takers (parents, educators, nurses) can give them if they suspect any discomfort or pain without any need of additional medical advice.
    a. Totally agree

   b. Agree
   c. Disagree
   d. Totally disagree
   e. Don't know

26. Children with PIMD's pain tends to be overtreated.
   a. Totally agree
   b. Agree
   c. Disagree
   d. Totally disagree

27. Children with PIMD's pain tends to be undertreated.
   a. Totally agree
   b. Agree
   c. Disagree
   d. Totally disagree

28. In what area do you face difficulties that prevent you from taking good care of a child with PIMD?

	Very important barrier	Important barrier	Barrier not important	Not a barrier
Pain assessment				
Communication with the child				
Personal experience				
Personal knowledge about pain				
Personal knowledge about children with disabilities				

29. What barriers do you perceive to pain medication in children with PIMD?

	Very important barrier	Important barrier	Barrier not important	Not a barrier
Fear of side effects				
Risk of addiction to analgesics				
Treatment can hide the source of pain				
Treatment can hinder the investigation of pain				
Risk of respiratory depression				
Fear of opioids				
Parental pressure				
